# MOC Doped with Graphene Nanoplatelets: The Influence of the Mixture Preparation Technology on Its Properties

**DOI:** 10.3390/ma14061450

**Published:** 2021-03-16

**Authors:** Martina Záleská, Milena Pavlíková, Adam Pivák, Šimon Marušiak, Ondřej Jankovský, Anna-Marie Lauermannová, Michal Lojka, Filip Antončík, Zbyšek Pavlík

**Affiliations:** 1 Department of Materials Engineering and Chemistry, Faculty of Civil Engineering, Czech Technical University in Prague, Thákurova 7, 166 29 Prague, Czech Republic; martina.zaleska@fsv.cvut.cz (M.Z.); milena.pavlikova@fsv.cvut.cz (M.P.); adam.pivak@fsv.cvut.cz (A.P.); simon.marusiak@fsv.cvut.cz (Š.M.); 2 Department of Inorganic Chemistry, Faculty of Chemical Technology, University of Chemistry and Technology, Technická 5, 166 28 Prague, Czech Republic; ondrej.jankovsky@vscht.cz (O.J.); Anna-marie.Lauermannova@vscht.cz (A.-M.L.); michal.lojka@vscht.cz (M.L.); filip.antoncik@vscht.cz (F.A.)

**Keywords:** magnesium oxychloride cement, graphene nanoplatelets, mixing method, structural parameters, mechanical properties

## Abstract

The ongoing tendency to create environmentally friendly building materials is nowadays connected with the use of reactive magnesia-based composites. The aim of the presented research was to develop an ecologically sustainable composite material based on MOC (magnesium oxychloride cement) with excellent mechanical, chemical, and physical properties. The effect of the preparation procedure of MOC pastes doped with graphene nanoplatelets on their fresh and hardened properties was researched. One-step and two-step homogenization techniques were proposed as prospective tools for the production of MOC-based composites of advanced parameters. The conducted experiments and analyses covered X-ray fluorescence, scanning electron microscopy, energy-dispersive spectroscopy, high-resolution transmission electron microscopy, sorption analysis, X-ray diffraction, and optical microscopy. The viscosity of the fresh mixtures was monitored using a rotational viscometer. For the hardened composites, macro- and micro-structural parameters were measured together with the mechanical parameters. These tests were performed after 7 days and 14 days. The use of a carbon-based nanoadditive led to a significant drop in porosity, thus densifying the MOC matrix. Accordingly, the mechanical resistance was greatly improved by graphene nanoplatelets. The two-step homogenization procedure positively affected all researched functional parameters of the developed composites (e.g., the compressive strength increase of approximately 54% after 7 days, and 37% after 14 days, respectively) and can be recommended for the preparation of advanced functional materials reinforced with graphene.

## 1. Introduction

Graphene is a carbon-based, two-dimensional nanomaterial and consists of a single monolayer of graphite with a thickness of one atom (0.34 nm). Due to its structure, graphene exhibits extraordinary physical properties such as a high specific surface area, ultrahigh tensile strength and modulus of elasticity, and excellent electrical, optical, and thermal parameters. Graphene nanoplatelets (GNPs) are composed of a few layers of graphene and have a total thickness of less than 100 nm. GNPs inherit many advantages of graphene and, in contrast to it, GNPs can be produced on a large-scale and are cheaper than carbon-based nanotubes and nanofibers [1,2,3]. The price of GNPs is about 400 EUR/kg, which is not high with respect to its low content in the composition of graphene-doped composites.

In recent years, the addition of GNPs to Portland cement-based composites to enhance, in particular, their mechanical, electrical, and durability-related properties was studied intensively. Wang et al. [4] reported that the incorporation of 0.05 wt. % of GNPs increased the flexural strength by 15–24%, and the compressive strength by 3–8% respectively, in the comparison with the plain cement paste. Liu et al. [5] investigated the effect of GNPs and graphene oxide nanoplatelets (GONPs) on the mechanical and piezoresistive properties of cementitious materials. Tao et al. [6] studied the influence of GNPs on microstructure, mechanical properties, and electric conductivity of cement mortar. The electrical potential method was used for the evaluation of the structural health of the GNPs-infused mortar in the study reported by Le et al. [7]. The enhancement of resistance against water and chloride ingress of concrete with the content of GNPs was researched by Du et al. [8]. Other approaches in the use of graphene and other carbon-based nanoadditives have been previously described in the literature [9,10,11,12].

As graphene is prone to aggregate in a variety of matrices, it is necessary to achieve the uniform dispersion and distribution of graphene in the mixture to fully manifest its excellent properties [13]. The problem of poor dispersion of graphene in silicate cementitious mixtures was investigated by many authors, and a variety of methods were suggested. The most commonly used graphene dispersion methods include ultrasonic treatment, application of ionic/non-ionic surfactants, and the addition of silica fume [14,15,16]. The problematics of the nanoadditive dispersion are further described in the literature [17,18,19].

As the requirements of the construction materials grow, the industry of Portland cement (PC) grows with them. The main scope of the development of PC and PC-based concrete is the improvement of its mechanical properties. Previous studies have shown various approaches in this area [20,21,22]. However, the process of the PC production is very burdensome for the environment. The production of Portland cement (PC) was globally 4.1 Gt in 2019, and this amount is directly related to the CO_2_ emissions generated in cement manufacturing, due to both the combustion of fuels and the decomposition of limestone in the clinker production process. This represents approximately 900 kg of CO_2_ released per ton of produced cement. To achieve the necessary cement sector decarbonization, high-performance alternative binding materials with low carbon footprints should be researched and put into construction practice [23,24]. Magnesium oxychloride cement (MOC) appears to be one of the suitable candidates due to (a) a lower calcination temperature during the production of caustic magnesium oxide (~750 °C) in contrast with ~1450 °C in the case of the Portland clinker, which moreover allows the use of alternative fuels, and (b) the fact that MOC-materials have the potential to sequestrate the CO_2_ from the atmosphere through carbonation process to form carbonates and hydroxycarbonates during their lifespan [25,26]. MOC is an aerial binder and is formed by mixing magnesium oxide (MgO) powder with a solution of magnesium chloride (MgCl_2_) at a specified ratio [27]. This ratio affects the formation of four main phases. At ambient temperature 3Mg(OH)_2_·MgCl_2_·8H_2_O (also called Phase 3) and 5Mg(OH)_2_·MgCl_2_·8H_2_O (Phase 5) phases can be prepared, which are responsible for the strength and hardness of MOC. At the temperature of ~100 °C, 2Mg(OH)_2_·MgCl_2_·4H_2_O (Phase 2) and 9Mg(OH)_2_·MgCl_2_·5H_2_O (Phase 9) phases are stable [28]. MOC exhibits a series of advantageous properties in comparison with the PC, which include high strength, fast setting time, fire resistance, resistance to abrasion, the ability to accommodate a large amount of various aggregates, low cost, and energy savings in the process of production. Today’s main applications of MOC in the construction industry are flooring, fire protection systems, wall insulation, and decorative purposes. Unfortunately, as MOC is a kind of air-hardening binder, a long exposure to water leads to its degradation (brucite formation) and subsequently to the loss of mechanical resistance [29,30].

Although Portland cement-based composites with the graphene addition are researched intensively, there are very few published studies dealing with the application of graphene in MOC-based materials. The coupled influence of graphene and diatomite on the properties of MOC-composites was recently reported [31]. The high-strength nanocomposites based on the MOC with the addition of graphene, graphite oxide, and a combination of both nanomaterials in the amount of 0.5 wt. % of binder were investigated [32]. To date, no study has been found to investigate the effect of MOC-graphene mixture preparation technology on the parameters of resulting composites.

Therefore, the main objective of this study is to evaluate comprehensively the influence of the MgCl_2_-GNPs aqueous suspension dispersion prior to the preparation of MOC-GNPs fresh mixture, and the additional homogenization of this fresh mixture after the ordinary mixing procedure on the basic structural, microstructural, and mechanical properties of the hardened composites. The obtained results will provide a basic framework for the design of MOC-GNP nanocomposites.

## 2. Materials and Methods

### 2.1. Chemicals

The composition of the investigated materials is given in Table 1. All MOC mixtures had the same composition of magnesia binder. The two major components of the control nanocomposite (MOC-REF) and MOC-graphene specimens (MOC-G and MOC-G-H) were light MgO powder and MgCl_2_·6H_2_O of p.a. purity (both obtained from Lach-Ner, Ltd., Neratovice, Czech Republic) dissolved in tap water. The chemical composition of MgO, analyzed by X-ray fluorescence (ARL QUANT’X EDXRF Spectrometer, Thermo Scientific, Madison, WI, USA), was 93.2 wt. % of MgO, 6.2 wt. % of Al_2_O_3_, and the residual amounts were only traces of SiO_2_, CaO, and Cl. Graphene nanoplatelets with a surface area of 300 m^2^·g^−1^ (Sigma Aldrich, St. Luis, MO, USA) were incorporated in 0.5% weight ratio of all dry substances used in the mixture. As shown in Table 1, this ratio was identical for both MOC-graphene mixtures. It is expected, due to the graphene doping, that the unit price of MOC-G materials will be around 0.75 EUR higher than that of pure MOC.

### 2.2. Synthetic Procedures

**Figure 1:**
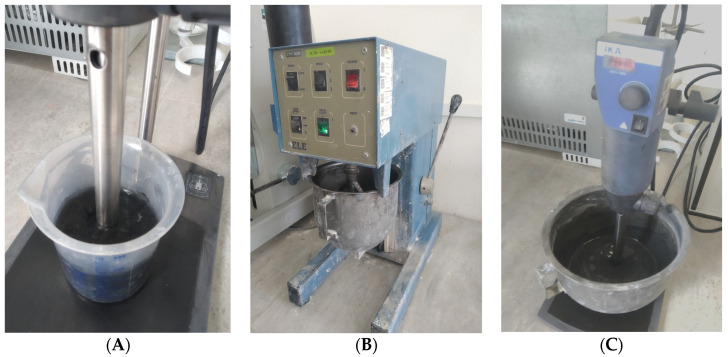
Preparation of composites: (**A**) dispersion of graphene in MgCl2 solution; (**B**) ordinary mixing of MOC (magnesium oxychloride cement) paste; (**C**) second step of homogenization using Ultra-Turrax.

For the evaluation of homogenization influence on the fresh and hardened paste properties, MOC-graphene composites were prepared with mixing methods based on one-step and two-step homogenization, respectively. Both methods started with the first step of homogenization, where graphene nanoplatelets were dispersed in MgCl_2_ solution using a mechanical rotor-stator homogenizer Ultra-Turrax T-18 (IKA, Staufen im Breisgau, Germany). This first mixing was conducted for 5 min at 10,000 rpm. After that, the ordinary mixing procedure based on the technical standard EN 14016-2 [23] was applied. Magnesia powder and MgCl_2_ solution for MOC-REF, or graphene-MgCl_2_ solution in the case of MOC-GNPs composites, respectively, were placed into a planetary type mortar mixer (ELE). The mixing started at a low speed regime (paddle 140 rpm, mixing head 62 rpm) for 1 min, followed by high-speed mixing (paddle 285 rpm, mixing head 125 rpm) for 0.5 min. Then, the mixing was stopped to clean the mixer head and the sides of the mixing bowl. After that, mixing continued for another 1 min at high speed followed by casting of MOC-REF and MOC-G samples. The second step of homogenization was applied for MOC-G-H samples only. This composite mix was additionally homogenized using a mechanical homogenizer for another 5 min at 10,000 rpm. The two-step preparation of composites is documented in Figure 1.

All samples were cast into steel molds and were exposed to a laboratory temperature of 23 ± 2 °C and relative humidity of 30 ± 5% for 4 h, followed by demolding and air-curing under the same laboratory conditions for 7 and 14 days, respectively.

### 2.3. Analytical Techniques

Morphology of the samples was studied by scanning electron microscopy (SEM), optical microscopy (OM), and high-resolution transmission electron microscopy (HR-TEM). The sample surface was determined by Brunauer, Emmett, and Teller (BET). The phase composition was studied via X-ray diffraction (XRD) and Fourier-transform infrared spectroscopy (FTIR). Rotation viscometry was used to measure the change in viscosity of fresh composite mixtures.

Among macrostructural parameters of the hardened composites, bulk density *ρ*_b_ (kg·m^−3^), specific density *ρ*_s_ (kg·m^−3^), and total open porosity *Ψ* (%) were measured. The microstructure of the formed composites was characterized by mercury porosimetry. Lastly, compressive strength *f*_c_ (MPa) and flexural strength *f*_f_ (MPa) were tested. The experimental details can be found in Supporting Information (SI). The expanded combined uncertainty of the applied test methods was as follows: *ρ*_b_ 1.4%, *ρ*_s_ 1.2%, *Ψ* 2.0%, *f*_c_ 1.4%, *f*_f_ 1.4%.

## 3. Results and Discussion

The microstructure of the typical graphene nanoplatelet provided by HR-TEM is shown in Figure S1 (SI). BET surface of graphene is introduced in Figure S2 (SI).

**Figure 2:**
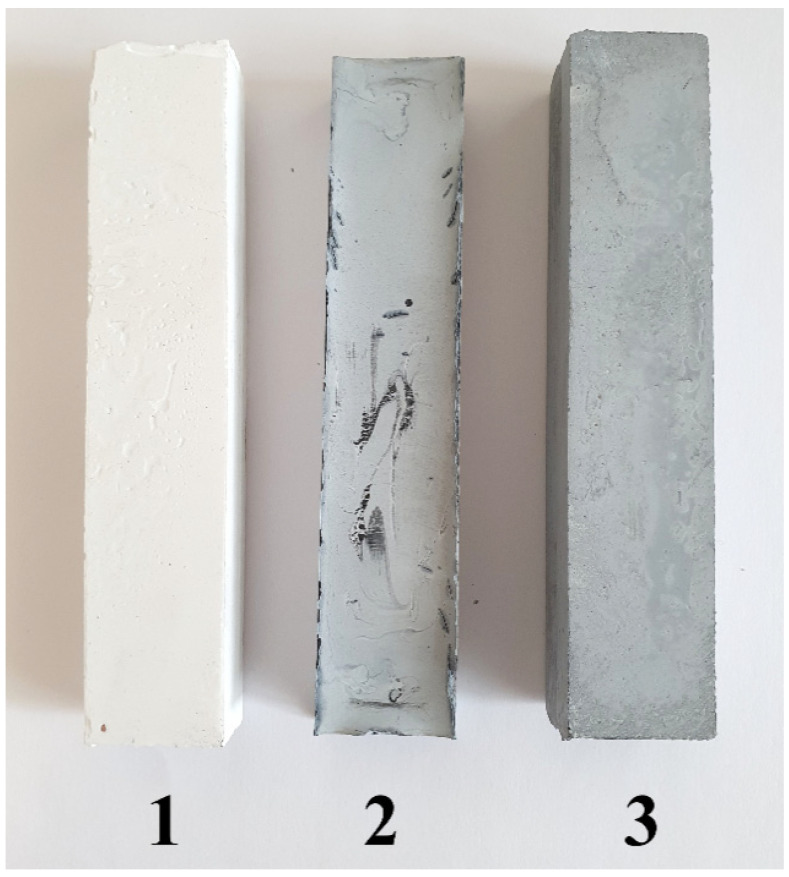
Photograph of prepared prismatic specimens of (**1**) MOC-REF (a reference sample with pure Magnesium Oxychloride Cement), (**2**) MOC-G (Magnesium Oxychloride Cement with Graphene), and (**3**) MOC-G-H (Magnesium Oxychloride Cement with Graphene Homogenized by Ultra-Turrax).

MOC-graphene composites and MOC reference without graphene were prepared. The samples were termed as MOC-REF (a reference sample with pure Magnesium Oxychloride Cement), MOC-G (Magnesium Oxychloride Cement with Graphene) and MOC-G-H (Magnesium Oxychloride Cement with Graphene Homogenized by Ultra-Turrax). The photograph of the prepared samples is shown in Figure 2.

**Figure 3:**
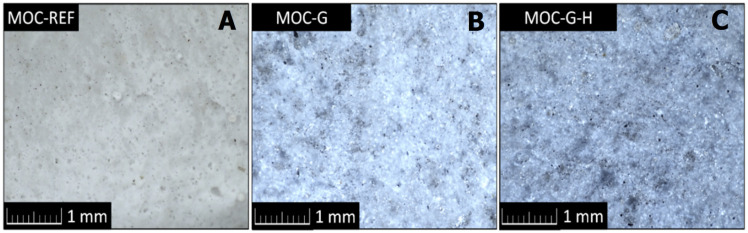
Optical micrographs of the prepared composites: (**A**) MOC-REF, (**B**) MOC-G and (**C**) MOC-G-H

The fracture surface of the composite samples was studied using optical microscopy. The micrographs (see Figure 3) show the difference between the composites containing graphene and the reference sample, where the color of the sample changes not only with the content of graphene but also with the rate of homogenization. The overall structure of the fracture surface is compact with no visible cracks or air bubbles.

**Figure 4:**
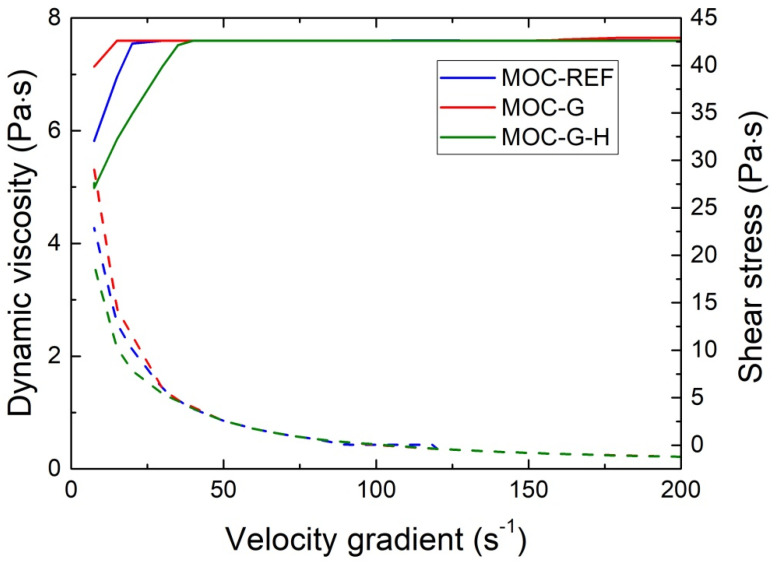
Shear stress and dynamic viscosity of the fresh composite mixtures.

The rheological curves obtained for fresh composite mixtures are graphed in Figure 4. The dependence of shear stress and velocity gradient conforms to the Bingham model of ideal plastic fluid [33] and for higher-velocity gradients to rheopectic fluid. For velocity gradient <50 s^−1^, the effect of the addition of graphene on viscosity and thus rheological performance was negligible, which is promising for the design and development of multi-component MOC composites enriched with nanoadditives. It can be expected that the admixing of fillers and mineral admixtures will decrease the viscosity of the composite mixtures. Therefore, the minimal influence of graphene nanoplatelets on the viscosity of examined composites is very beneficial. For lower-velocity gradients, the differences between the viscosity and shear stress of the tested fresh mixtures were well apparent. They can be assigned to the continuous precipitation of MOC constituents. In this case, the lowest shear stress, and thus the dynamic viscosity, exhibited the well homogenized mixture MOC-G-H. This is a positive finding that proves the applicability of the proposed homogenization technique in the production of MOC composites doped with graphene.

**Figure 5:**
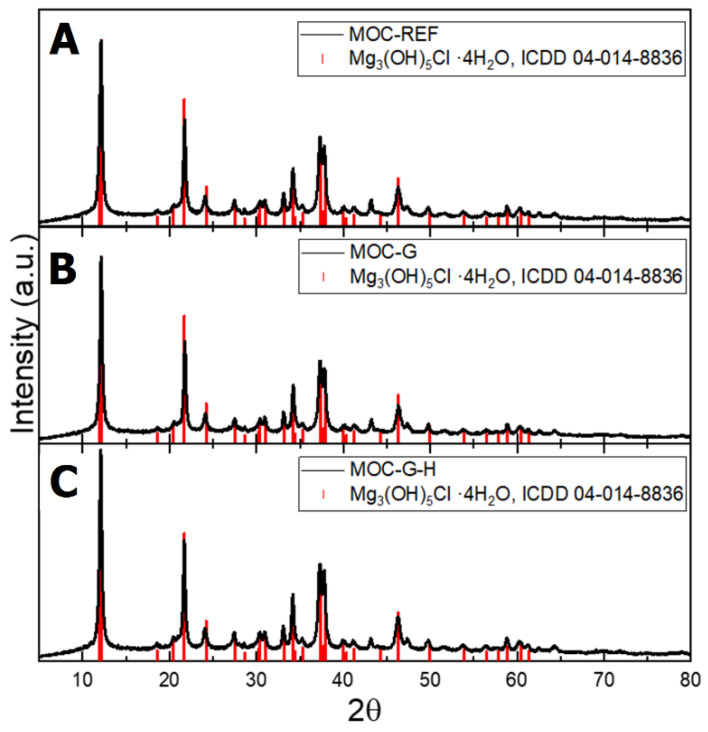
The diffraction patterns of the samples: (**A**) MOC-REF, (**B**) MOC-G, and (**C**) MOC-G-H.

The phase composition of the 14-day samples was analyzed using XRD. The obtained diffraction patterns are shown in Figure 5. The only crystalline phase present in the pattern of all the samples was Mg_3_(OH)_5_Cl·4H_2_O (ICDD 04-014-8836), which corresponds with the stoichiometry of MOC Phase 5. Graphene was not visible in the diffraction patterns due to its very low content in the composites. Due to that, the diffraction patterns of all three samples were quite similar.

**Figure 6:**
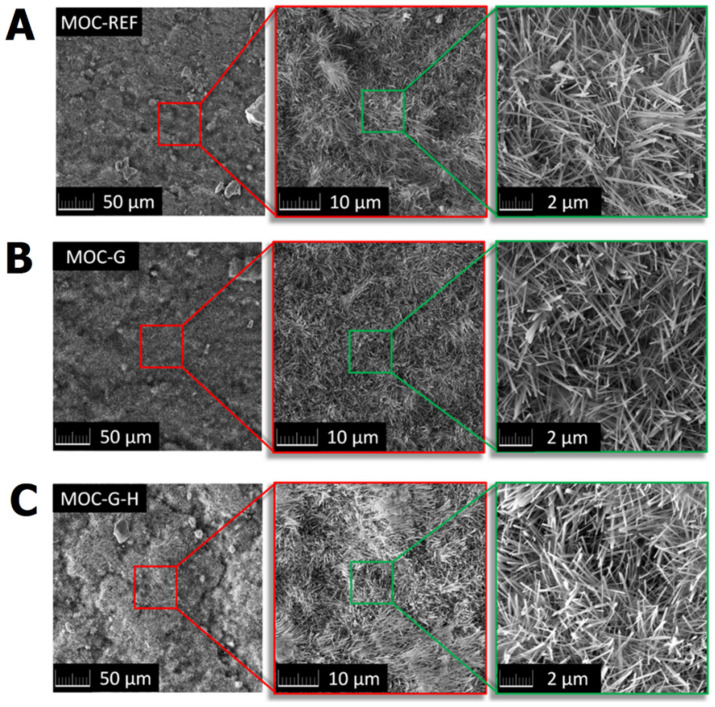
Micrographs of prepared samples: (**A**) MOC-REF, (**B**) MOC-G, and (**C**) MOC-G-H.

The microstructure of MOC-REF and prepared MOC composites combined with graphene was analyzed using SEM. The SEM micrographs of the prepared composites are shown in Figure 6. The prepared samples showed the presence of needle-shaped crystals typical for MOC-based materials. The typical dimensions of the needles were 1–6 µm in length and ~0.5 µm in thickness. Graphene was not visible because micrographs were obtained from the fracture surface. It was previously confirmed that graphene significantly improves the mechanical properties (flexural strength and compressive strength) [32]; therefore, the fracture occurred in areas with low graphene concentration.

**Figure 7:**
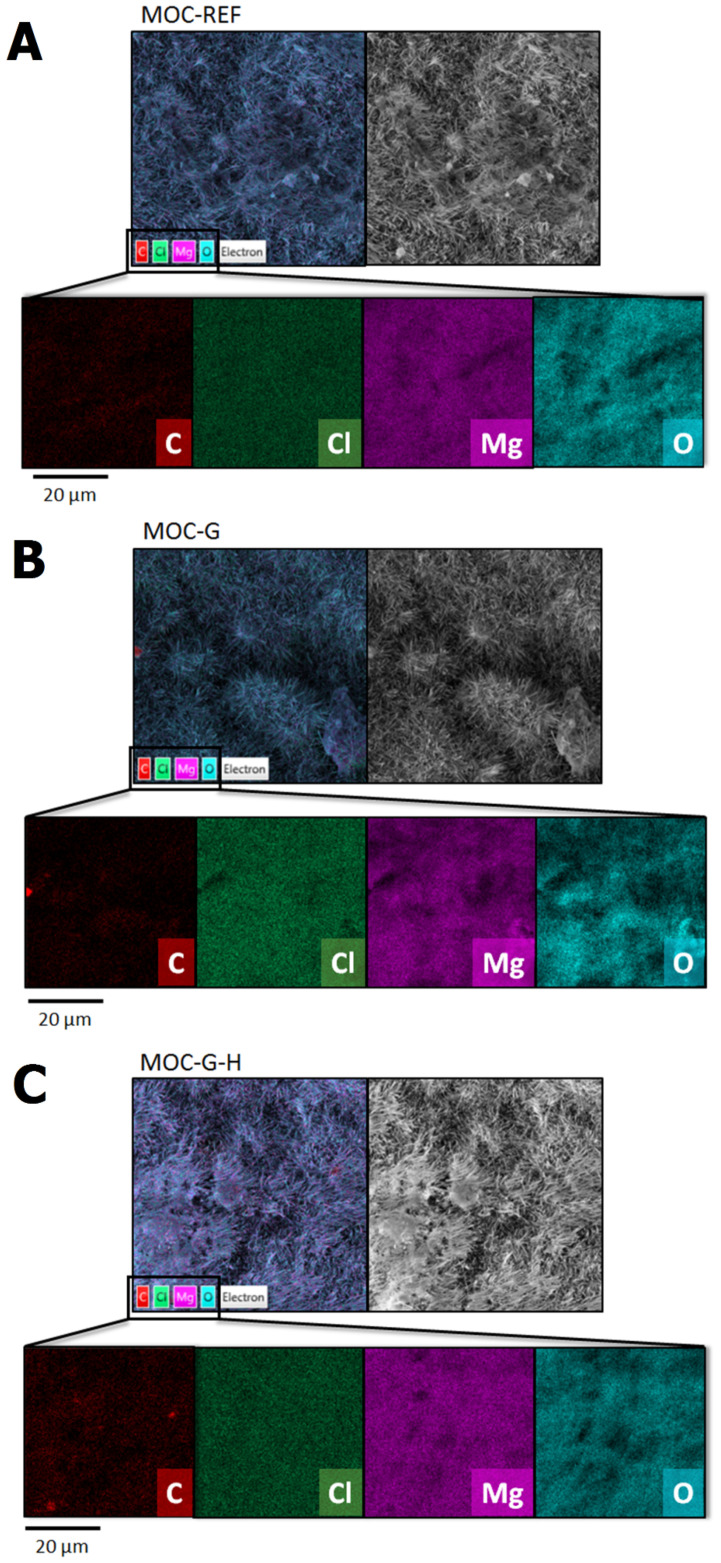
EDS elemental maps of samples: (**A**) MOC-REF, (**B**) MOC-G, and (**C**) MOC-G-H.

The EDS elemental maps were measured to obtain the chemical composition for comparison of reference and prepared composites. EDS detected the presence of Mg, O, Cl, and C and confirmed the high purity of the prepared samples. Obtained composition was similar for all samples corresponding to the stoichiometry of pure Phase 5. All elemental maps are displayed in Figure 7. Relatively higher carbon content was detected, most probably due to the surface reaction with carbon dioxide forming chlorartinite [34].

**Figure 8:**
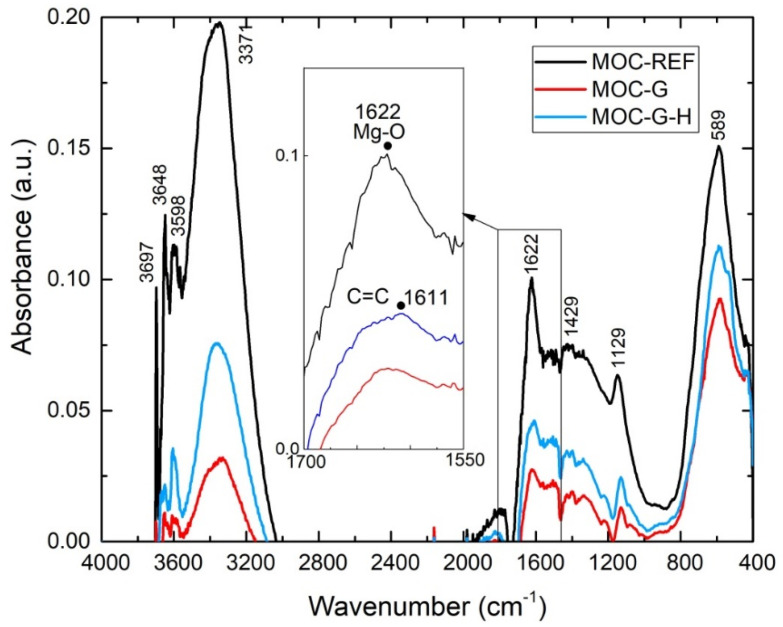
The MIR spectrum of MOC 5-1-8 phase in the range of 400–4000 cm^−1^.

The collected FT-IR spectra are presented in the form of spectral lines in Figure 8 and as a summarization of the major absorption band assignments in Table 2. All analyzed samples contained the bands coming from the fundamental vibrations of structural H_2_O, and the lattice vibrations of MgCl_2_ and Mg(OH)_2_. The absorption bands between 3700 and 3600 cm^−1^ are caused due to stretching modes of O-H bonds in the hydroxyl group in Mg(OH)_2_, while the broad absorption band with maximum at 3375–3346 cm^−1^ can be assigned to the stretching vibration of O-H bonds in the hydroxyl group in water. The band intensities at 1646, 1608, and 1157 cm^−1^ are bending vibrations of O-H bonds in the hydroxyl group in Mg(OH)_2_ [35]. At 1622 and 1429 cm^−1^, the stretching vibration of Mg-O in MgCl_2_·8H_2_O can be found. The series of modes in the range of 1000–400 cm^−1^ originated from the lattice translation vibrations (Mg-OH) and vibrational vibrations of Mg-O/Mg^2+^, O/O-Mg-O/O-Mg^2+^-O bonds [36]. Lattice vibration modes of Mg-O/Mg-Cl bonds around 500 cm^−1^ can be contributed by the vibrations of the bonds of Mg-Cl or Mg-O.

The analyzed spectrum of graphene evidences only one weak band appearing at 1586 cm^−1^, which can be attributed to the stretching vibration of the C=C bond [37,38,39]. In the case of MOC composites this band is shifted to higher wave numbers, specifically at 1611 cm^−1^. This band also partially overlaps with the intensive band of the stretching vibration of Mg-O in MgCl_2_·8H_2_O. Based on this phenomenon the peak resolution was found to be at 1611 cm^−1^ in the case of MOC-G and MOC-G-H samples.

The macrostructural parameters of the hardened composites are shown in Table 3. In both curing ages, the influence of the graphene addition on the drop in porosity was visible. Moreover, the advanced mixing method enabled a further decrease in the porosity accessible for helium molecules [40]. The drop in porosity measured for 14-day cured samples was 21% for MOC-G and 33% for MOC-G-H, respectively, which clearly characterized the effectiveness of the proposed homogenization procedure. The differences in the bulk density and specific density were small, but in comparison with the reference material, the bulk density was typically higher for graphene-doped composites; contrary to that, the specific density was reduced by the incorporation of the nanoadditive into the composite mixture.

**Figure 9:**
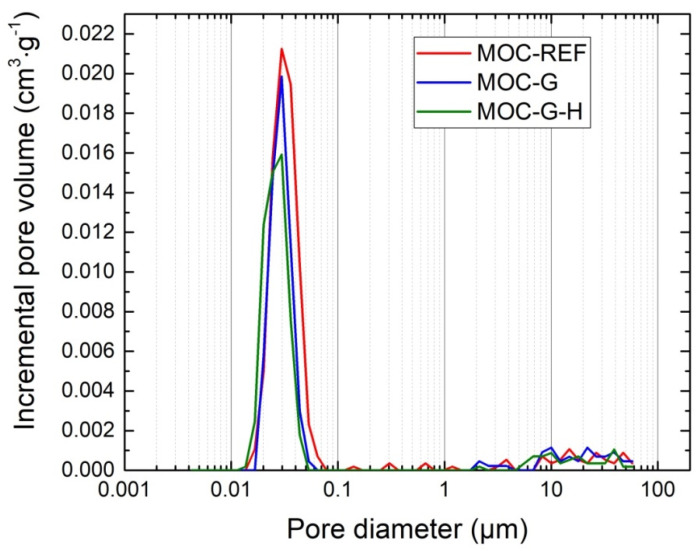
Incremental pore volume distribution for 14-day composites.

**Figure 10:**
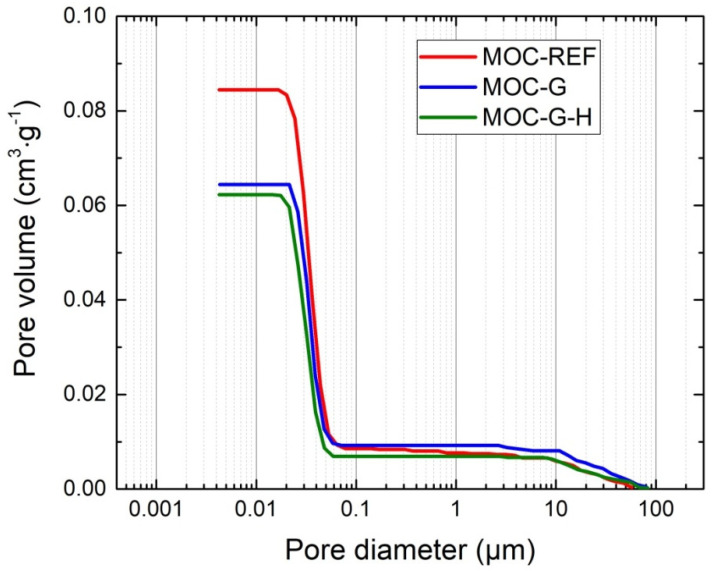
Cumulative pore volume distribution for 14-day composites.

The pore size distribution curves obtained by mercury intrusion porosimetry are given in Figure 9 and Figure 10. Apparently, the microstructural data correspond with the total porosity data presented in Table 3. The differences between the total porosity data obtained by MIP and calculated on the basis of the bulk density and specific density values are small, especially considering the samples’ size and principals of the applied techniques.

The microstructural parameters of 14-day cured samples are summarized in Table 4. The pore volume visibly decreased with the addition of graphene, but the average pore size was slightly higher for graphene-enriched materials, compared to the reference composite MOC-REF. This was due to the increase in the volume of pores in the diameter range 100–10 μm, and the decrease in the volume of nanometer-sized pores (5–35 nm).

**Figure 11:**
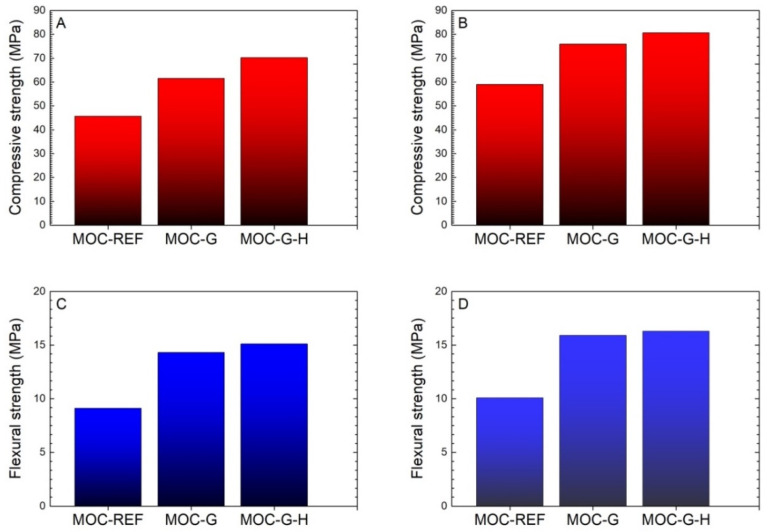
Mechanical parameters: (**A**) compressive strength of 7-day samples; (**B**) compressive strength of 28-day samples; (**C**) flexural strength of 7-day samples; (**D**) flexural strength of 28-day samples.

The investigated mechanical parameters are presented in Figure 11. As a potential construction material, MOC composites exhibited high compressive strength and flexural strength as similarly presented, e.g., by Misra et al. [41], Góchez et al. [42], and most recently Lauermannová et al. [43]. The effect of graphene addition on the mechanical resistance of the developed composites is well apparent. The use of the nanoadditive in the form of graphene nanoplatelets greatly improved both the compressive and flexural strength, which corresponded well with the total porosity data and pore size distribution. In comparison with the reference composite MOC-REF, the increase in the compressive strength was remarkable in both curing ages. For the 7-day samples, the increase in the compressive strength was 34.6% for MOC-G, and 53.6% for MOC-G-H, respectively. In the case of 14-day matured samples, the increase in the compressive strength values was 28.6% and 36.6% for MOC-G and MOC-G-H composites, respectively. Similarly, for the 7-day samples the enhancement of the flexural strength was 57.1% for MOC-G and 65.9% in the case of the MOC-G-H composite. The 14-day samples showed an increase in the flexural strength of 57.4% for MOC-G and 61.4% for MOC-G-H.

The two-set homogenization procedure led to the increased growth of the compressive strength. On the other hand, the improvement of the flexural strength due to the modified homogenization procedure was not great, but still sufficient for the practical use of graphene-reinforced MOC materials in construction practice. Obviously, the two-step homogenization procedure improved the distribution of graphene particles in MOC fresh mixture, which consequently resulted in the upgraded parameters of the hardened composites.

For all researched composites, the prolonged curing time significantly increased both observed mechanical parameters. This is in agreement with the findings of other authors, who reported the strength development of MOC mixtures with curing age [44]. This was due to the solidification of the precipitated MOC phases, i.e., the free intergrowth of crystal phases and, thus, densifying of their microstructure [45]. Nevertheless, in the estimation of the mass transport in the researched materials, and thus their durability, one must consider the fact that the porosity essentially affects the mechanical strength, whereas the transport processes depend on the structure and the size distribution of the pores [46].

## 4. Conclusions

The effect of the mixture homogenization procedure in the preparation of magnesium oxychloride cement paste doped with graphene nanoplatelets on its fresh and hardened properties was studied. The addition of graphene and the process of two-step homogenization resulted in the following aspects:

1. The addition of graphene did not increase the viscosity of the fresh mixtures, which is positive for their presumed application in the design and development of the advanced types of multi-component MOC composites for construction applications.
2. Both presented mixing procedures designed for MOC mixtures with graphene enabled the production of materials with decreased porosity and greatly improved mechanical resistance, in comparison with the ordinary MOC paste.
3. The two-step mixing technique even improved the structural and mechanical parameters acquired for MOC-graphene composite homogenized using the one-step technology.

This represents very promising results for the future research and prospective application of carbon-based nanoadditives in the production of building materials.

## Figures and Tables

**Table 1:** Composition of tested paste samples.

Mixture ID	Mass (g)			
	Magnesia Powder	MgCl_2_·6H_2_O	H_2_O	Graphene
MOC-REF	900	908.4	562.1	-
MOC-G	900	908.4	562.1	9.042
MOC-G-H	900	908.4	562.1	9.042

**Table 2:** Assignments of the major absorption bands of MOC composites.

Wavenumber (cm^−1^)	Assignment
3697, 3648, 3598	stretching (ν) vibration of O-H in Mg(OH)_2_
3375–3346	stretching (ν) vibration of H-O-H in H_2_O
1646, 1608, 1157	bending (δ) vibration of H-O-H in MgCl_2_·8H_2_O
1622, 1429	stretching (ν) vibration of Mg-O in MgCl_2_·8H_2_O
1611	stretching (ν) vibration of C=C
844	stretching (ν) vibration of Mg-O cubic structure
668, 634	stretching (ν) vibrations of Mg-O
589	deformation (δ) and stretching (ν) lattice vibrations of Mg-Cl/Mg-O
535	translation vibrations of Mg/Mg-O, Mg-OH
440	vibrational modes of the lattice showing the Mg-O/Mg^2+^, O/O-Mg-O/O-Mg^2+^-O bonds

**Table 3:** Basic structural parameters of the hardened composites.

Material	7-Day Aged Samples			14-Day Aged Samples			
	*ρ*_b_ (kg·m^−3^)	*ρ*_s_ (kg·m^−3^)	*Ψ* (%)	*ρ*_b_ (kg·m^−3^)	*ρ*_s_ (kg·m^−3^)	*Ψ* (%)	*Ψ_Hg_*^1^ (%)
MOC-REF	1603	1867	14.2	1633	1887	13.5	13.4
MOC-G	1680	1854	11.2	1658	1857	10.7	10.4
MOC-G-H	1654	1848	10.5	1652	1818	9.1	9.8

^1^ Total porosity measured by MIP (Mercury Intrusion Porosimetry).

**Table 4:** Microstructural parameters of the 14-day composites.

Parameter	MOC-REF	MOC-G	MOC-G-H
Total pore volume (cm^3^·g^−1^)	0.0844	0.0644	0.0622
Average pore diameter (µm)	0.0295	0.0317	0.0320

## Data Availability

The data presented in this study are available on request from the corresponding author. The data are not publicly available due to privacy.

## Supplementary Materials

The following are available online at https://www.mdpi.com/1996-1944/14/6/1450/s1, Figure S1: Microstructure of used graphene obtained by TEM (**A**); detail of graphene obtained by higher magnification (**B**). Figure S2: Specific surface area of graphene obtained by BET method.
