# Supplementary material for: MOC Doped with Graphene Nanoplatelets: The Influence of the Mixture Preparation Technology on Its Properties
Source: Materials (Basel). 2021 Mar 16;14(6):1450. doi: 10.3390/ma14061450 (PMC8002252; doi:10.3390/ma14061450)
Supplement: Supplementary file 1 [file materials-14-01450-s001.pdf]

materials

MDPI

Supplementary Material

MOC Doped with Graphene Nanoplatelets: The Influence of the Mixture Preparation Technology on its Properties

Martina Záleská [1], Milena Pavlíková [1], Adam Pivák [1], Šimon Marušiak [1], Ondřej Jankovský [2], Anna-Marie Lauermannová [2], Michal Lojka [2], Filip Antončík [2] and Zbyšek Pavlík [1,*]

[1] Department of Materials Engineering and Chemistry, Faculty of Civil Engineering, Czech Technical University in Prague, Thákurova 7, 166 29 Prague, Czech Republic; martina.zaleska@fsv.cvut.cz (M.Z.); milena.pavlikova@fsv.cvut.cz (M.P.); adam.pivak@fsv.cvut.cz (A.P.); simon.marusiak@fsv.cvut.cz (Š.M.)
[2] Department of Inorganic Chemistry, Faculty of Chemical Technology, University of Chemistry and Technology, Technická 5, 166 28 Prague, Czech Republic; ondrej.jankovsky@vscht.cz (O.J.); Anna-marie.Lauermannova@vscht.cz (A.-M.L.); michal.lojka@vscht.cz (M.L.); filip.antoncik@vscht.cz (F.A.)
* Correspondence: pavlikz@fsv.cvut.cz; Tel.: +420-224-354-371

To study the surface morphology of the samples, scanning electron microscopy (SEM) performed on a Tescan MAIA 3 (Tescan Brno, Brno, Czech Republic) was used. The elemental composition and maps were obtained using an energy dispersive spectroscopy (EDS) analyzer (X-Max150) with a 20 mm² SDD detector (Oxford Instruments, Oxfordshire, UK) and AZtecEnergy software (software version 3.0, Oxford instruments, HighWycombe, UK). The sample was held on a carbon conductive tape in order to ensure the conductivity of the experiments. For both SEM and SEM-EDS analysis, the electron beam was set to 10 kV, with 10 mm work distance.

High-resolution transmission electron microscopy (HR-TEM) was performed using an EFTEM Jeol 2200 FS microscope (Jeol, Tokyo, Japan). A 200 keV acceleration voltage was used for the measurement. The sample preparation was attained by drop-casting the suspension (1 mg mL^{-1} in water) on a TEM grid (Cu; 200 mesh; Formvar/carbon) and then drying in a vacuum dryer at 25 °C and $p/p^0 = 0.2$. The obtained microstructure of typical graphene nanoplatelet is shown in Figure S1.

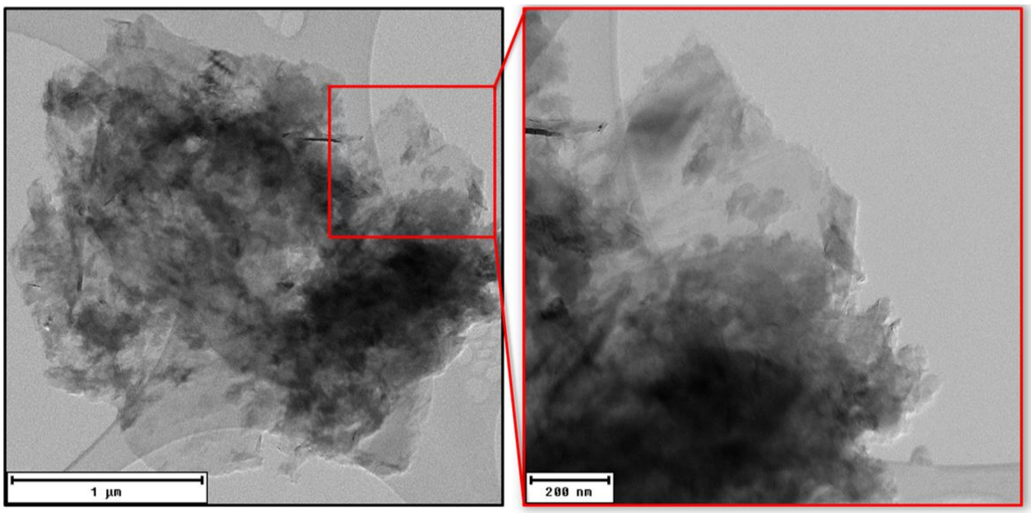

(**A**) (**B**)

Figure S1. Microstructure of used graphene obtained by TEM (**A**); detail of graphene obtained by higher magnification (**B**).

Publisher's Note: MDPI stays neutral with regard to jurisdictional claims in published maps and institutional affiliations.

Copyright: © 2021 by the authors. Licensee MDPI, Basel, Switzerland. This article is an open access article distributed under the terms and conditions of the Creative Commons Attribution (CC BY) license (http://creativecommons.org/licenses/by/4.0/).

Additionally, BET specific surface was measured using a sorption analyzer NOVAtouch LX2 (Quantachrome Instruments, Boynton Beach, FL, SUA). The sample was outgassed for 10 h at 50 °C under high vacuum. The reason for such a low temperature is to avoid degradation and further decomposition of oxygen functionalities. Nitrogen cooled (77 K) detector was used for the evaluation of the results using BET (Brunauer, Emmett and Teller) and Kelvin equations. For dry and degassed sample was used 11 points BET measurement to obtained more accurate isotherm. The Quantachrome software version 1.2. (Quantachrome Instruments, Boynton Beach, FL, SUA) was used for evaluation of measured data and to recalculate the measured value to m^2 per 1 gram. The measured isotherm of used graphene is shown in Figure S2. Calculated surface area was 302.7 $m^2 \cdot g^{-1}$ which is in good agreement with the declared surface area by manufacturer.

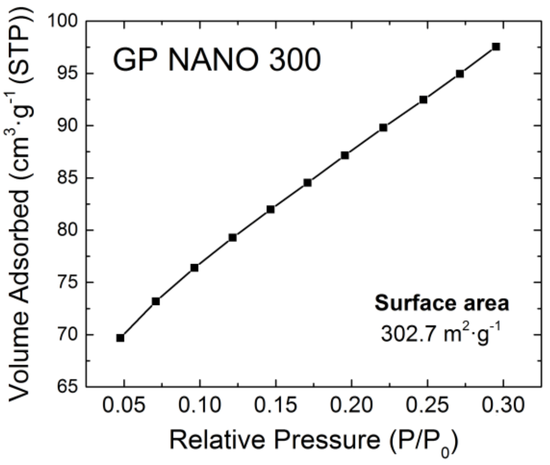

Figure S2. Specific surface area of graphene obtained by BET method.

X-ray powder diffraction (XRD) was carried out by Bruker D2 Phaser (Bruker, Karlsruhe, Germany), and powder diffractometry was employed with Bragg–Brentano geometry, applying CuK_α radiation (λ = 0.15418 nm, U = 30 kV, I = 10 mA) and sample rotation (5 rpm). The step size was set to 0.02025° (2θ), and the overall data were collected from the angular range of 5–80°. Measurements were performed after 1 week of curing.

To validate the presence and incorporation of graphene in MOC composites, the FT-IR spectroscopy was employed. Using Nicolet 6700 spectrometer (Thermo Fisher Scientific, Waltham, MA, USA), the mid infrared spectra were collected after 32 scans. The wavenumbers ranged from 4 000 to 400 cm^{-1} and a spectral resolution was 4 cm^{-1}. Firstly, dry samples were crushed and consequently homogenized with a ball grinder MM 400 (RETSCH, Haan, Germany). The mid infrared spectra were analyzed on a diamond crystal using ATR technique.

Optical microscopy of composite samples was performed by a Navitar macro-optics (Rochester, NY, USA) microscope with optical zoom up to 110X and recorded with digital camera Sony 2/3" having a resolution of 5Mpix. The sample was illuminated by a white LED ring light source with individually addressable segments and intensity. NIS-Elements BR 5.21.02 software (Laboratory Imaging s.r.o., Prague, Czech Republic) with an Extended Depth of Focus Module (EDF) was used for imagining and analysis of the samples.

The changes in the viscosity of the fresh composite mixtures due to the addition of graphene nanoplatelets were monitored by a rotation viscometer HAAKE Viscotester E (Thermo Fisher Scientific, Waltham, MA, USA).

The hardened composites were tested at the age of 7 and 14 days, respectively. Among the macrostructural parameters of the hardened composites, bulk density ρ_b

($kg{\cdot}m^{-3}$), specific density ρ_s ($kg{\cdot}m^{-3}$), and total open porosity Ψ (%) were measured. For each composite, five samples were tested. The bulk density was evaluated from the specimen volume and its mass as prescribed in the EN 1015-1. The specimens were prisms with dimensions 40 mm × 40 mm × 160 mm. As the bulk density tests were finished, the specimens were pounded and the fragments were used for the measurement of specific density which was done using a helium pycnometer Pycnomatic ATC (Porotec, Bensheim, Germany). The porosity was calculated based on the bulk density and specific density data. The microstructure of the tested materials was investigated using set of mercury porosimeters of Pascal series, Pascal 140 and Pascal 440 (Thermo Fisher Scientific, Waltham, MA, USA). In the evaluation of mercury intrusion porosimetry (MIP) data, the circular cross section of capillaries was assumed. The typical dry sample mass in MIP test was about 1.0 g. The tested mechanical parameters were flexural strength f_f (MPa) and compressive strength f_c (MPa). The strength tests were realized in accordance with the EN 1015-11. In the three point bending test, the flexural strength was measured on the standard prisms. For each material, 4 samples were tested. The compressive strength was measured on the fragments from the flexural strength test. The compression force was applied on the 40 mm × 40 mm cross section of the specimens. In total, 5 samples were submitted to the compressive strength measurement.
